# Review of Oral Rabies Vaccination of Dogs and Its Application in India

**DOI:** 10.3390/v14010155

**Published:** 2022-01-14

**Authors:** Gowri Yale, Marwin Lopes, Shrikrishna Isloor, Jennifer R. Head, Stella Mazeri, Luke Gamble, Kinzang Dukpa, Gyanendra Gongal, Andrew D. Gibson

**Affiliations:** 1 Mission Rabies, Panjim 403002, India; 2 Department of Animal Husbandry & Veterinary Services, Government of Goa, Panjim 403001, India; marwinlopes@gmail.com; 3 Bangalore Veterinary College, Hebbal, Bengaluru 560024, Karnataka, India; kisloor@gmail.com; 4 Division of Epidemiology, University of California Berkeley, Berkeley, CA 94720, USA; jennifer_head@berkeley.edu; 5 The Roslin Institute, The Royal (Dick) School of Veterinary Studies, The University of Edinburgh, Easter Bush Veterinary Centre, Midlothian, Roslin EH25 9RG, UK; stella.mazeri@roslin.ed.ac.uk (S.M.); andy@missionrabies.com (A.D.G.); 6 Mission Rabies, Dorset, Cranborne BH21 5PZ, UK; Luke@missionrabies.com; 7 World Organisation for Animal Health (OIE), Regional Representation for Asia and the Pacific, Tokyo 113-8657, Japan; k.dukpa@oie.int; 8 World Health Organization (WHO), Regional Office for South East Asia, New Delhi 110002, India; gongalg@who.int

**Keywords:** oral rabies vaccine, free roaming dogs, dog mediated human rabies, canine rabies control

## Abstract

Oral rabies vaccines (ORVs) have been in use to successfully control rabies in wildlife since 1978 across Europe and the USA. This review focuses on the potential and need for the use of ORVs in free-roaming dogs to control dog-transmitted rabies in India. Iterative work to improve ORVs over the past four decades has resulted in vaccines that have high safety profiles whilst generating a consistent protective immune response to the rabies virus. The available evidence for safety and efficacy of modern ORVs in dogs and the broad and outspoken support from prominent global public health institutions for their use provides confidence to national authorities considering their use in rabies-endemic regions. India is estimated to have the largest rabies burden of any country and, whilst considerable progress has been made to increase access to human rabies prophylaxis, examples of high-output mass dog vaccination campaigns to eliminate the virus at the source remain limited. Efficiently accessing a large proportion of the dog population through parenteral methods is a considerable challenge due to the large, evasive stray dog population in many settings. Existing parenteral approaches require large skilled dog-catching teams to reach these dogs, which present financial, operational and logistical limitations to achieve 70% dog vaccination coverage in urban settings in a short duration. ORV presents the potential to accelerate the development of approaches to eliminate rabies across large areas of the South Asia region. Here we review the use of ORVs in wildlife and dogs, with specific consideration of the India setting. We also present the results of a risk analysis for a hypothetical campaign using ORV for the vaccination of dogs in an Indian state.

## 1. Introduction

The ancient disease of rabies continues to spread unchecked in the free-roaming dog populations across much of the developing world. Whilst examples of elimination through mass dog vaccination stretch back a century [1], these methods are yet to be implemented at the scale needed to control the rabies virus in much of Africa and Asia. India is estimated to account for 35% of global human rabies deaths, approximating 21,000 deaths a year, causing an annual loss of 2.3 billion USD through premature death, bite treatment, loss of labor, livestock losses and post-exposure prophylaxis [2]. 

Whilst considerable progress has been made to increase accessibility to post-exposure prophylaxis in India [3], the continued circulation of rabies virus in the dog population will inevitably result in human exposures and its health and economic implications for generations to come [4]. India has the highest estimated incidence of human and canine rabies globally, and a large population of free-roaming dogs. Achieving the global 2030 target set by the Tripartite (Food and Agriculture Organization (FAO), World Organisation for Animal Health (OIE), World Health Organization (WHO)) and the Global Alliance for Rabies Control (GARC) of zero human rabies deaths due to canine rabies will require the coordinated, systematic and sustained annual vaccination of millions of dogs in India for many years [5,6]. However, the large inaccessible dog population typical of many settings in India presents a considerable logistical challenge for the scaling-up of parenteral vaccination strategies [7]. 

The use of oral rabies vaccination (ORV) in dogs has been proposed for the past 30 years [8], and has been the foundational tool for the elimination of rabies virus from wildlife species across the world for over 50 years [9,10]. Today there is overwhelming support from global institutions, including WHO and OIE, for the operational evaluation of ORV of dogs in rabies-endemic settings to complement parenteral approaches [11]. Developing effective scalable approaches to mass dog vaccination in India will not only progress rabies control in one of the most severely affected countries, but would also generate momentum for the uptake of similar approaches in the South Asia region.

In this review we consider aspects of mass dog vaccination and ORV pertinent to rabies control in India and present a risk analysis for a hypothetical campaign using the oral-bait-handout approach in Goa state, India.

## 2. The Need of ORV of Dogs in India

### 2.1. Achieving Herd Immunity in an Inaccessible Population

There are three commonly used parenteral vaccination strategies for mass dog vaccination: static point (SP), door-to-door (DDV) and capture-vaccinate-release (CVR) [12]. The efficacy of each method at achieving high vaccination coverage in the target dog population is dependent upon the composition of that population with regard to ownership and accessibility [13].

SP vaccination involves vaccination teams establishing temporary dog vaccination clinics at which dogs are brought by dog owners for vaccination. This method has been used to great effect at a continental scale in Latin America where 50 million dogs were vaccinated during a single week every year [14,15]. High vaccination coverages have also been reported in a number of SP campaigns in Africa [16,17]. This approach requires a high degree of community engagement, a large proportion of the dog population being owned and those owners being willing and able to bring their dogs to clinics for vaccination. In areas where SP turnout is insufficient to achieve high vaccination coverage, DDV can boost coverage by sending vaccination teams of one or two people through communities visiting each household and vaccinating dogs that can be held for parenteral vaccination [12,18].

Whilst the SP or DDV approaches are able to achieve high dog vaccination coverage in many endemic settings, they are unlikely to access sufficiently high proportions of the population in areas where a large number of dogs are difficult or impossible to restrain by hand for parenteral vaccination [13]. Therefore more advanced techniques of net catching are required through the CVR method to reach annual dog vaccination coverages approaching 70% [19]. Whilst effective, CVR has high fixed operational costs (salaries, vehicles, equipment) and requires a large, skilled workforce [20]. Additionally, it can exacerbate an increasingly inaccessible dog population as dogs become wary of catching teams. 

The issue of high CVR fixed operational costs can be somewhat offset by incorporating DDV as the primary method to efficiently vaccinate dogs readily available for restraint by hand, followed by CVR to access the inaccessible population [19,20]. This does not, however, overcome the major operational limitation of the need to manage a huge skilled workforce focused entirely on sustaining the annual vaccination of dog populations across vast geographic areas. The complex contact structure of dog populations requires vaccination campaigns to be coordinated and synchronized, incorporating urban, peri-urban and rural settings in which enzootic rabies virus transmission is sustained [21]. Existing parenteral methods have been used for the systematic annual vaccination of hundreds of thousands of dogs where additional expertise and resources are available but would be infeasible across the larger states of India to control rabies. 

### 2.2. Competing Priorities for Dog Population Management

When it comes to stray dogs, concerns over rabies are generally superseded by other perceived issues caused by roaming dogs, including barking, road traffic accidents and hygiene [22]. Although this emphasizes that a broader awareness of rabies is greatly needed, the issue of stray dog population management will remain a public and political priority. Given the large evidence base showing that dog culling is ineffective at sustaining dog population reduction and is often detrimental to rabies control [23,24], many governments are seeking sustainable solutions through large-scale dog sterilization as a part of humane dog population management interventions. Dog population management is a far more complex undertaking than mass dog vaccination, requiring well-managed surgical veterinary infrastructure, improvement in public services such as waste disposal, as well as a broad shift in dog ownership culture, dog abandonment and dog reproductive control [25]. Whilst investigations of less labor-intensive tools for dog sterilization are ongoing, there is a need to evaluate the impacts of existing surgical approaches more comprehensively [26].

The juxtaposition between the desire to control canine reproduction through surgical sterilization and the need for pan-societal annual dog vaccination to achieve rabies control has the potential to limit the impact of both. ORV would enable vaccination of difficult-to-catch dogs without the need to handle them, thus allowing for the intensive annual vaccination of stray populations without impacting the likelihood of being able to capture dogs for surgical sterilization later as a part of dog population management efforts.

## 3. Types of ORV

**Figure 1:**
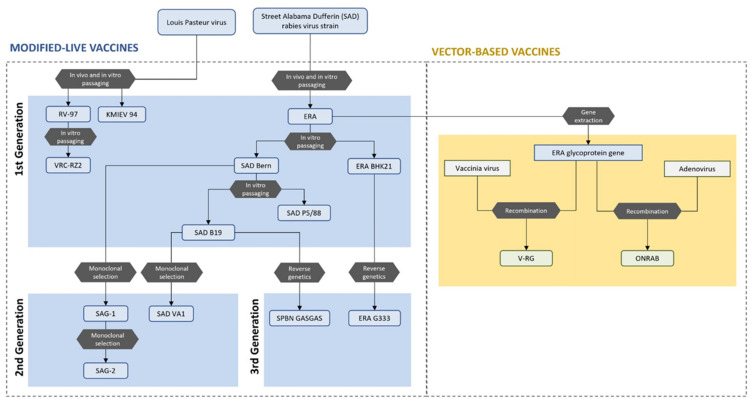
Diagram showing the parental derivation of ORVs that have been licensed for use in wildlife in countries within Europe, Asia or North America. First generation modified-live vaccines were derived from SAD rabies virus strain in 1935. Second and third generation of modified-live vaccines were developed through monoclonal selection and reverse genetics in 1980s and 1990s. Vector-based vaccines were developed from ERA strain by gene extraction and recombination with vaccinia and adenoviruses in the 1980s.

There are two types of ORV currently being used under commercial license for the vaccination of various wildlife species; these are modified live vaccines (MLVs) (also called attenuated live rabies virus vaccines) and vector-based vaccines (VBVs) (Figure 1). The active component of MLVs is live, replication-competent rabies virus that has been modified so that it no longer causes disease, but still induces the body’s natural immune response [27]. In contrast, VBVs are created by inserting antigenic glycoprotein encoding genetic material from the rabies virus into other vector viruses, which then express rabies virus glycoprotein within the vaccinated individual, inducing an immune response.

### 3.1. Modified Live Vaccines (MLVs)

Almost all modified live rabies virus vaccines in use today are derived from a single rabies virus strain, named Street Alabama Dufferin (SAD), isolated by the CDC in the USA in 1935. This strain underwent extensive passaging through non-neural cell lines (hamster kidney, pig kidney cells and embryonated chicken eggs) and thermal stabilization to varying degrees to form a range of highly attenuated ORVs, including SAD-Bern, ERA and SAD-B19. This first generation of ORVs were the foundation of rabies control in Europe and remain the most widely used ORVs globally [28].

The safety profile of first-generation ORVs was improved by inducing selection mutations using monoclonal antibodies, resulting in the creation of the second generation ORVs, including SAG1 and SAG2 [29]. The development of 3rd generation MLVs has resulted from modern technologies of reverse genetics, which have enabled site-directed mutagenesis, targeting specific changes at selected locations in the rabies virus genome. These site-specific deletions and insertions further improve the safety and immunogenicity of existing MLVs. There are two 3rd generation vaccines currently tested for use in canids: SPBN GAS GAS and ERA G333 [11,30]. Although originating from different parent vaccine virus strains, both vaccines have similar mutations at residue 333 of the G-protein [28].

Attenuated live rabies virus vaccines rely on mutations to the rabies glycoprotein gene, which is a major contributor to viral pathogenicity through its role in viral uptake, budding and avoiding neuronal impairment [31,32,33]. The vaccine virus is taken up in palatine tissue where it undergoes limited local replication, however modifications to the vaccine virus prohibit normal pathogenic mechanisms and increase apoptosis [34]. Limited local replication of the virus occurs within the oral tissues generally inducing a strong and life-long immunity against the rabies virus due to exposure to the full range of rabies virus antigens. The vaccine virus does not shed in the urine or feces due to destruction in the gastrointestinal tract, however, it can be detected in the oral cavity for several hours after consumption [35,36].

The primary concern with using MLVs is the potential for reversion to virulence through natural random mutation of the virus, enabling the vaccine virus to regain its capacity to cause rabies [37]. The complicating consequences of this have been widely documented following the large-scale use of MLVs in the global campaign for polio eradication, with ongoing challenges due to the circulation of vaccine-derived polio viruses [38]. The sustained distribution of MLVs for rabies control in wildlife throughout the world for over 40 years provides a robust evidence base on which to study this risk for ORVs. The marker of safety for attenuated-live rabies virus vaccines is the ability to induce rabies following intracerebral inoculation into immunocompromised mice [39]. First generation MLVs have been shown to still be capable of causing rabies following intracranial inoculation in immunosuppressed mice [40]. Eleven cases of vaccine-associated rabies were reported in immunosuppressed foxes and non-target species in Europe following vaccination with first generation MLVs, representing an incidence of 1 in 48 million bait doses distributed [29]. Onward transmission of vaccine-derived rabies virus was not detected and so these cases had no epidemiological significance [41]. Furthermore, no cases of field reversion to pathogenicity of second or third generation MLVs have been reported. No adverse events associated with human contacts with MLV ORVs have ever been reported.

### 3.2. Vector-Based Vaccines (VBVs)

Vector-based ORVs were developed to avoid the theoretical risks associated with the use of live rabies virus vaccines. VBVs are created through the insertion of a segment of cDNA encoding the rabies virus glycoprotein into the genome of a vector virus, which is subsequently expressed within the vaccinated individual [42]. Two VBVs are currently commercially licensed for use in wildlife, both of which express the rabies virus glycoprotein; RABORAL V-RG, which uses recombinant vaccinia virus (*Orthopoxvirus* genus) as the vector [35], and ONRAB, which uses recombinant human adenovirus 5 as the vector [43,44] (Figure 1).

One of the problems encountered with the use of VBVs is the potential for disease caused by the vector virus. Human exposure to V-RG has been associated with severe skin inflammation and there have been reports of complications in pregnant and immunocompromised individuals in USA [45,46]. Another disadvantage of VBVs is the potential interference by pre-existing immunity against the vector, which may inhibit uptake and generation of sufficient immunity against rabies [43,47]. Therefore it is possible that the efficacy of campaigns using VBVs may be hindered in settings where a large proportion of the animal population has immunity against the vector virus [48]. This is a concern for vaccines with adenovirus as the vector, as it is ubiquitous in many areas. 

Over 1 billion ORV bait doses have been distributed in North America and Europe over the past four decades, predominantly across large forest areas through helicopter and airplane distribution methods (Table 1) [28]. The first ORV field trials were conducted in Switzerland in 1978 using modified live ORV to explore the rabies control in the red fox (*Vulpes vulpes*) population [27]. After successful pilots and expanded implementation in other European countries through the 1980s [49,50], the European Union (EU) committed financial support for national ORV campaigns in Member States from 1989 [51,52]. Continued development of ORVs resulted in more than ten commercially licensed attenuated and recombinant ORVs used in the EU, including SAD Bern, SAD B19, SAG1, SAG2 and V-RG [29,50]. Over the past four decades, more than 736 million ORV baits have been distributed across 30 countries in Europe, covering an area of 2.75 million km^2^ [51], equivalent to the area of India’s 17 largest states combined. 

In contrast to the red fox rabies reservoir in Europe and Canada [53], enzootic rabies virus transmission is sustained in skunks (*Mephitis mephitis*) and racoons (*Procyon lotor*) in many parts of North America [1,54]. Differences in the oropharyngeal anatomy of these species means that uptake of MLVs in immunogenic tissues is less effective as compared to canid species, resulting in reduced efficacy of these vaccines [34,55,56]. VBVs, however, have been demonstrated to produce an effective immune response and have been used extensively to control rabies in these non-canid wildlife populations [57]. Table 1 delineates ORVs used in wildlife and trialed in dogs.

**Figure 2:**
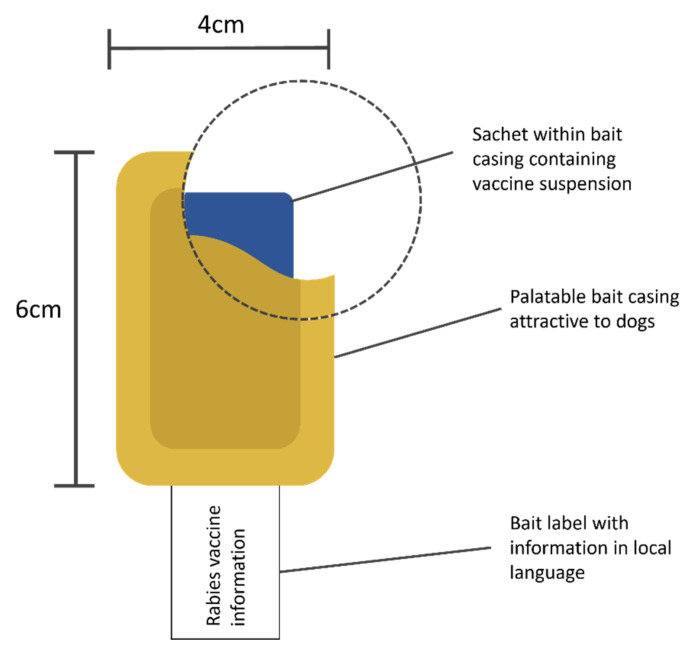
Illustration of components of an example ORV bait construct for use in dogs. The dotted circle shows a cut-away to reveal the impermeable sachet containing vaccine suspension within bait casing. Information is generally either printed directly on the bait casing or as a protruding label.

ORVs are administered orally via a bait construct (Figure 2). The bait construct size and composition is specific to target species to suit, taste preference and eating behavior [58]. Both MLVs and VBVs are held in liquid suspension at a volume and concentration appropriate to the target species. The vaccine suspension is placed within a sealed sachet which is then cased in a palatable bait material specific to the target species. Upon bait consumption the sachet is perforated by the action of chewing, causing the vaccine suspension to be released in the oral cavity. Here, the vaccine is taken up predominantly by the palatine tonsils where it induces a protective immune response after limited replication at the site of entry [9].

## 4. Oral Rabies Vaccination of Dogs

As in wildlife species, the effective vaccination of dogs using ORV requires a bait construct designed to maximize uptake and release of the vaccine suspension in the oral cavity. Aspects of smell, taste, texture and size are all likely to play a role in palatability and inducing chewing to perforate the vaccine sachet. Baits which are too small and are swallowed without perforating the sachet will not be immunized. Numerous studies have performed to evaluate preference for different bait casing materials, including fishmeal, animal intestine, chicken head, egg and dog food based materials [59,60,61,62,63,64,65]. There was considerable variation in the preference for different bait materials by location, possibly reflecting the normal diet of the population. Studies in India, Bangladesh and Thailand all reported high rates of uptake and perforation in egg-based bait constructs [59,63,66]. Egg-based bait constructs also have the benefit of being broadly culturally acceptable and offering potential for mass-production with basic production facilities.

The oral-bait-handout method has been described for the distribution of ORV in urban settings [20,67]. In this approach, a vaccination team of two people travelling by bike can simultaneously conduct parenteral vaccination of dogs that can be handled for injection, whilst distributing baits to dogs considered infeasible to handle. Baits are tossed from a distance to dogs selected for ORV, taking care not to startle the dog. The dog is observed whilst the bait is consumed and any unconsumed baits, vaccine packaging and bait remnants are collected and disposed safely by the vaccination team. The cost effectiveness and feasibility of this model has been demonstrated in Goa [20], Haiti [67], Morocco [68], USA [62], Tunisia [69,70], Turkey [71], Philippines [72], Guatemala [73] and Sri Lanka [74].

## 5. Evaluation of Available ORVs for Use in Dogs

The formal process of vaccine licensure requires a national regulatory authority to review the safety and efficacy of a product in target species, non-target species and humans. This is invariably an expensive and complex process, often requiring studies of ethical concern. The development of global human health initiatives in the 1970s required assurance of consistent standards of vaccine quality, safety and efficacy, however it was infeasible to seek licensure in every country of use [88]. This challenge was navigated through the development of the WHO vaccine prequalification programme in 1987, which evolved over several decades to provide international standards of vaccine safety and efficacy for use in national immunization programs [88].

Such mechanisms are yet to be developed for the veterinary vaccine sector, however there is broad consensus on the need for international scrutiny of ORVs to make the required evidence readily accessible to national regulatory authorities for decisions of ORV implementation [11]. The CDC, OIE, WHO and others recently published updated recommendations for the evaluation of ORV candidates considered for field use [11,89]. Table 2 outlines these recommendations and provides a review of the currently available ORVs.

Off-label use of vaccines is common, even for large scale national immunization initiatives, where safety and efficacy has been demonstrated, but licensure has not been completed [90]. Therefore, although the WHO and OIE have emphasized the need to continue licensure processes, they also emphasize that this should not be considered a prerequisite to conducting field evaluations of ORVs deemed to be safe and effective [11].

### 5.1. Safety Risk Analysis

Many ORVs have undergone the stringent process of licensure for use in wildlife in North America and Europe. Such authorization through national regulatory agencies such as US Department of Agriculture Center for Veterinary Biologics or the European Medicines Agency include comprehensive assessment of human safety in the context of distribution in areas proximal to human habitats. 

The CDC developed a Markov chain model to evaluate the human safety of environmental distribution of ORV for vaccination of dogs [135]. Simulations were conducted for hypothetical dog ORV campaigns in an average rabies-endemic country, excluding China and India, using SAD B19 (1st generation MLV) and SPBN GASGAS (3rd generation MLV). Whilst the simulation using the 1st generation MLV, SAD B19, estimated 3.35 human deaths per 10 million baits distributed, no human deaths were predicted from the simulation using the 3rd generation MLV, SPBN GASGAS [135].

To evaluate the human safety of a hypothetical dog vaccination campaign in India, we applied the model using local parameters from Goa State. The simulated campaign consisted of the distribution of 40,000 baits of SPBN GASGAS over a 12-day period in urban cities and towns as well as rural villages. The full report of this analysis is provided in the supplementary materials (Table S1) and summarized here.

We performed two analytical methods: the first being a standard analysis, using predicted parameters based on local data sources and published literature, and the second, a sensitivity analysis, using Latin Hypercube sampling from the distribution of each possible parameter values (Supporting Materials). For both analyses, we ran 1000 simulations, and calculated the mean across simulations. We calculated the 95% confidence interval as the 2.5th and 97.5th percentile of the simulations. 

The standard analysis estimated that a mean of 32,006 dogs would be vaccinated and that no dogs or non-target animals would get vaccine-induced rabies. A mean of 5.1 human exposures were estimated (95% CI: 0, 14; Range: 0, 20), resulting in 4.0 extra health care visits (95% CI: 0, 11; Range: 0, 16), but no serious adverse events (SAE) of human deaths during the campaign (Range: 0, 0), as well as no human deaths when extrapolated per 10 million baits distributed (Table 3). In all simulations of this population per 10 million baits distributed, no dogs or non-target animals were expected to get vaccine-induced rabies. The most common exposure expected was bites from dogs that had recently consumed the vaccine (Table 4). 

Simulations from the sensitivity analysis predicted a mean of 27,919 dogs and 201 non-target animals would be vaccinated and no dogs or non-target animals would develop vaccine-induced rabies. The model predicted a mean of 4.9 human exposures resulting in 3.4 extra health care visits, no SAEs, and no human deaths. Values from the standard analysis are expected to be more accurate than the simulated outputs from the sensitivity analysis due to the use of predicted parameters based on available data (Supporting Materials). Nevertheless, the range of values from the sensitivity analysis is important as it represents the span of outcomes across the potential parameter space. In the worst-case simulation, there were 24 exposures, 22 health care visits, no SAEs and no deaths. In the best-case simulation, there were no exposures, health care visits, SAEs or human deaths (Table 3). Of the simulated exposures, there was a range of 0 to 1 for severe bites, 0 to 4 for licks from recently vaccinated animals and 0 to 24 for bites from recently vaccinated animals (Table 4).

### 5.2. Candidate ORV for India

It is likely that ORV will be required to complement parenteral dog vaccination campaigns in both rural and urban settings. The simulation analysis predicted low rates of human exposure through the hand-out method in such areas; however vaccine safety remains of paramount importance especially due to the close dog–human relationship in India.

The prediction of no severe adverse events and no human deaths per 10 million SPBN GASGAS bait distributions reflects the robust safety profile of this 3rd generation MLV. SPBN GASGAS resulted from site-directed mutations to the SAD-B19 vaccine strain, altering all three nucleotides at amino acid positions 194 and 333 of the glycoprotein gene, reducing the likelihood of natural random mutation resulting in a reversion to virulence. The insertion of a second identical modified glycoprotein gene further enhances the safety profile of the vaccine over first and second generation MLVs. The attenuating effect of the insertion of additional genes is believed to be due to the reduced expression in downstream genes, which subsequently decrease the rate of replication of the virus in vitro and in vivo, as opposed to dominance of the non-pathogenic glycoprotein gene [32,136,137,138]. 

SPBN GASGAS has been shown to effectively generate an immune response in dogs comparable to parenteral vaccination, with regards to rabies virus neutralizing (RFFIT) and binding (ELISA) antibodies [84]. The high efficacy can be attributed to its high affinity to monocytes and immature dendritic cells [139]. The vaccine has also been shown to be safe and effective in other species including the small Indian mongoose, red fox, raccoon dog and raccoon under experimental conditions [109,110,140]. The vaccine has undergone numerous safety tests and is currently undergoing pilot field tests in several countries including Namibia and Nigeria.

SPBN GASGAS has been shown not to be shed in the environment for prolonged periods following the vaccination of an animal. The virus does not continue to replicate in the vaccinated animal and so there is an insignificant risk of onward transmission to other non-target animals or people and no risk of circulation of the virus in the environment [36]. The vaccine virus has been shown to be highly genetically stable and is non-pathogenic following intracranial inoculation into immune-competent mice [141].

Rabitec®, the trade name for SPBN GASGAS, is currently licensed for use in foxes and raccoon dogs in the European Union [100], and under consideration for use in the United States to vaccinate mongoose, however authorization from state and national authorities for importation and use in India is required. A practical challenge with using SPBN GASGAS in tropical and subtropical settings is its relatively low stability at high temperatures (above 20 °C). The proposed oral bait handout method for vaccine distribution would mean that vaccine would be dropped to individual dogs for immediate consumption, thereby limiting environmental exposure. Nonetheless, maintaining an effective cold-chain would be critical to the success of large scale vaccination campaigns using ORV, including freezer storage and insulated cool boxes for field transport. Planning campaigns for the cooler months of the year and distributing bait at cooler times of the day may also help to minimize the detrimental impact of temperature on the vaccine [139].

## 6. Conclusions

Oral rabies vaccination of dogs has been shown to improve the coverage of mass vaccination efforts in situations where dogs cannot be readily restrained or caught for parenteral vaccination. The long-standing and increasing support for the implementation of pilot field activities using modern ORVs from the WHO, OIE and other international experts provides reassurance to national authorities in their assessment of the suitability of these products. With the highest estimated incidence of human and canine rabies globally and a free-roaming dog population of tens of millions of dogs, India requires an efficient and effective operational solution to the mass vaccination of dogs that cannot be readily handled. Whilst the benefits of parenteral vaccination make it crucial for the vaccination of the accessible dog population, complementary use of ORV has the potential to enable the rapid scaling of high-coverage campaigns reaching the free-roaming reservoir population.

Based on our experience with mass dog vaccination in numerous states of India, including Goa, Tamil Nadu, Karnataka, Jharkhand, West Bengal and Maharashtra, the benefit of ORV as a complementary tool to parenteral vaccination methods to hasten the control of canine rabies is clear. Oral rabies vaccine is a strong tool that India is missing while striving to reach the Zero by 30 target to eliminate dog-mediated human rabies by 2030.

## Figures and Tables

**Table 1:** List of ORV used in wildlife and trialed in dogs.

Type	Vaccine Strain	Vaccine Name and Manufacturer	WILDLIFE					DOG		
			Species	Years In Use	Doses Distributed	Countries In Which Distribution Took Place	References	Year	Countries in Which Trials Have Taken Place	References
**Modified Live** **(1st generation)**	**SAD Bern**	Lysvulpen, Bioveta, Czech Republic	Red Fox, racoon dog	1979–1980	211,000,000	Europe	[29]	1994	Tunisia	[75]
	SAD B19	Fuchsoral, Ceva, France	Red fox	1978–2014	268,000,000	Europe	[29]	2001	Philippines	[72]
								1998	Turkey	[76]
	RV-97	Sinrab, FGBI ARRIAH, Russia	Racoon dogs	2002–current	4200,000	Kazakhstan, Ukraine, Belarus, Russia	[29,77]	-	-	-
	VRC-RZ2	Kazakhstan laboratory	Corsac fox, steppe wolf	2017	Laboratory	Kazakhstan	[78]	2017	Kazakhstan (laboratory)	[78]
	KMIEV-94	Institute of Experimental Veterinary, Belarus	Red fox	2009	10,300,000	Belarus	[29,79]	-	-	-
**Modified Live** (2nd generation)	SAG 2	RABIGEN® Virbac, France	Red fox, raccoon dog	199 –2012	28,000,000	France, Switzerland, Finland, Estonia, Italy, Germany, Belgium	[29,80]	2007	India	[81]
								1998	Tunisia	[82]
								2012	Morocco	[68]
**Modified Live** (3rd generation)	SPBN GASGAS	Rabitec® Ceva, France	Red fox, raccoon dog	201 -2019	Laboratory	Germany	[83]	2017	Haiti	[67]
								2020	Thailand	[84]
	ERA G333	Prokov, Russia	Red fox, raccoon dog	2017	Laboratory	Russia	[85]	-	-	-
**Vector–based** (Vaccinia virus)	V-RG	Raboral V-RG® Boehringer Ingelheim, Germany	Raccoon, coyote, grey fox, red fox, golden jackal, raccoon dog	1987–2017	250,000,000	USA, Canada, France, Belgium, Luxembourg, Ukraine, Israel, South Korea	[35]	2000	Sri Lanka	[86]
								2005	USA (laboratory)	[47]
**Vector-based** (Adenovirus)	AdRG1.3	ONRAB® Artemis Technologies Inc., Canada	Striped skunk, red fox, raccoon	2007–2017	28,500,000	Canada, USA	[28,44]	2016	USA (laboratory)	[61]
								2007	China (laboratory)	[87]

**Table 2:** Recommendations outlined by WHO and World Organisation for Animal Health expert committee on the suitability for field trials in dogs, and reference supporting fulfilment of that recommendation for each of the oral rabies vaccines currently used in wildlife. In addition to these criteria are five further considerations which are not listed as they are not specific to a vaccine. These are as follows: “Is the community supportive of oral rabies vaccination of dogs?”, “Can the responsible authority conduct postvaccination monitoring for persons potentially exposed to the vaccine?”, “Can the responsible authority conduct postvaccination monitoring for vaccine exposures from contact with recently vaccinated dogs?”, “Is there an effective postexposure prophylaxis for humans exposed to the oral rabies vaccine?”, “Can the responsible health authority provide postexposure prophylaxis for persons potentially exposed to the vaccine?”.

No.	Major Categories for Assessment of an Oral Rabies Vaccine Candidate	Modified Live Vaccines								Vector-Based Vaccines	
		SAD Berne	SAD B19	RV-97	VRC-RZ2	KMIEV-94	SAG 2	SPBN GASGAS	ERA G333	V-RG	AdRG1.3
1	Description of the manufacturer	[91]	[92]	[93]	-	-	[94]	[92]	-	[95]	-
2	Description of the vaccine construct	[96,97]	[98]	[77]	-	[79]	[99]	[100]	-	-	[101,102]
3	Is the vaccine safe for the target animal?	[75]	[103]	-	[78]	-	[82,104]	[67,105]	-	[47]	[106]
4	Has safety been assessed for potential non-target animals?	Jackals [107]	[103]	-	-	-	[108]	[83,109,110]	[85]	[35]	[106,111]
5	Has safety been assessed in nonhuman primates?	[112]	[113]	-	-	-	[114]	Conducted in parent vaccine SAD-B19 [113]	-	[115]	-
6	Does the vaccine elicit an immune response in target animals (dogs)?	[75]	[76]	-	[78]	-	[81,116]	[67,84,105]	-	[47]	[60,61,106]
7	Have virulent challenge studies been conducted to assess duration of immunity?	[117,118]	Foxes [119]	-	[78]	-	[116,120]	Foxes [121]	Foxes and raccoon dogs [85]	[35,122]	[106]
8	Does the vaccine replicate in host tissues and is replicating virus excreted from animals?	-	[103]	-	-	-	[104]	[36]	-	[123]	[106,124]
9	Is the bait composition attractive to the target animal, and does it convey delivery of the vaccine to the target host-anatomy?	-	-	-	-	-	[68]	[63,105]	-	-	-
10	Have bait contact rates been described for the bait distribution method you are considering?	-	-	-	-	-	-	[20,67,105]	-	-	-
11	Has the vaccine been evaluated under field conditions and are storage requirements known?	[125]	[126]	-	-	[127]	[128]	[67,105]	-	[129,130]	[131]
12	Has an economic cost-benefit assessment been conducted?	-	--	-	-	-	-	[20]	-	-	-
13	Is the product currently acknowledged by an international public health agency for field use?	[132]	_	_	_	_	_	[67,105]	_	[133]	_
14	Is the product currently licensed in any countries for field use?*	Europe	Europe	Russia	Kazakhstan	Belarus	[99] Europe	[134] Europe	Russia	Europe, USA	[102] Canada

* Licensure refers to wildlife only.

**Table 3:** Table of estimated exposures, health care visits and deaths from simulation of a 40,000 bait ORV campaign using estimated parameters for Goa, India. Standard analysis used parameters estimated from available data. Sensitivity analysis used Latin Hypercube parameter selection from the range of possible values.

	Value	Total Exposures	Total Health Care Visits	Total Human Deaths
**Standard analysis**	Mean (95% CI) per 40,000 baits	5.06 (0, 14)	3.98 (0, 11)	0 (0, 0)
	Rate per 10 million baits	1264	995	0
	Range per 40,000 baits	0–20	0–16	0–0
**Sensitivity analysis**	Mean (95% CI) per 40,000 baits	4.9 (0, 14)	3.4 (0, 12)	0 (0, 0)
	Range per 40,000 baits	0–24	0–22	0–0

**Table 4:** Types of exposures estimated from a 40,000 bait ORV campaign using estimated parameters for Goa, India.

		Contact with Baits		Interaction with Recently Vaccinated Animals (Dogs)			
	Value	Mucosal Contact	Transdermal Contact	Licks	Bites	Severe Bites	Bites from Rabid Animal
**Standard analysis**	Mean (95% CI) per 40,000 baits	0 (0, 0)	0 (0, 0)	0 (0, 1)	5.05 (0, 14)	0 (0, 1)	0 (0, 0)
	Rate per 10 million baits	0	0	1.25	1262.5	0.25	0
	Range per 40,000 baits	0–0	0–0	0–1	0–20	0–1	0–0
**Sensitivity analysis**	Mean (95% CI) per 40,000 baits	0 (0, 0)	0 (0, 0)	0.21 (0, 1)	4.98 (0, 11)	0 (0, 1)	0 (0, 0)
	Range per 40,000 baits	0–0	0–0	0–4	0–24	0–1	0–0

## Data Availability

All data used in the study is included in the text and Supplementary Materials.

## Supplementary Materials

The following supporting information can be downloaded at: https://www.mdpi.com/article/10.3390/v14010155/s1, Table S1: Parameter values used to evaluate the human safety of a 40,000 bait ORV campaign for dogs in Goa, India.
