# Supplementary material for: Review of Oral Rabies Vaccination of Dogs and Its Application in India
Source: Viruses. 2022 Jan 14;14(1):155. doi: 10.3390/v14010155 (PMC8777998; doi:10.3390/v14010155)
Supplement: Supplementary file 1 [file viruses-14-00155-s001.zip › viruses-1503727-supplementary.pdf]

**Table S1.** Parameter values used to evaluate the human safety of a 40,000 bait ORV campaign for dogs in Goa, India.

| | **Value** | **Lower bound** | **Upper bound** |
|---|---|---|---|
| **Vaccine Specific parameters for SPBN GASGAS** | | | |
| Probability a SAE will occur in an immune competent person who has made contact with the bait on a mucosal membrane (direct or via lick) | 0.00009 | 0.0000009 | 0.009 |
| Probability a SAE will occur in an immune competent person who has made contact with the bait transdermally (direct or via bite) | 0.00009 | 0.0000009 | 0.009 |
| Probability a SAE will occur in an immune competent person who has received a severe bite from an animal who has recently consumed the bait | 0.00009 | 0.0000009 | 0.009 |
| Probability a SAE will occur in an immune compromised person who has made contact with the bait on a mucosal membrane (direct or via lick) | 0.0378 | 0.011 | 0.378 |
| Probability a SAE will occur in an immune compromised person who has made contact with the bait transdermally (direct or via bite) | 0.0142 | 0.00142 | 0.142 |
| Probability a SAE will occur in an immune compromised person who has received a severe bite from an animal who has recently consumed the bait | 0.0779 | 0.0366 | 0.779 |
| Percent of animals who shed the vaccine in their saliva | 0.2 | 0.1 | 0.3 |
| Length of time virus can be shed in the saliva of an animal that has recently consumed the bait (days) | 0.5 | 0.16667 | 1 |
| Length of time the virus stays viable when left in the environment (days) | 4 | 1.5 | 15 |
| Probability an animal will experience a vaccine induced rabies case | 0 | 0 | 0 |
| Death rate of a rabid animal | 0.071429 | 0.05 | 0.1 |
| Probability someone with a severe bite that penetrates the cranial cavity or abdomen will not benefit from PEP | 0.7 | 0.6 | 0.8 |
| Probability that someone who receives PEP for any exposure other than a severe bite will not benefit from PEP | 0 | 0 | 0 |
| **Region-specific parameters** | | | |
| What proportion (0 to 1) of the population is severely immunocompromised? | 0.001 | 0.0001 | 0.01 |
| What proportion of the baits are expected to be ingested by the target animal? | 0.8 | 0.4 | 1 |
| What proportion of the baits are expected to be ingested by the non-target animal? | 0.005 | 0.0025 | 0.0075 |
| What proportion *of the unconsumed baits* are expected to be left in the environment? | 0.1 | 0.05 | 0.15 |
| How often will humans come into contact with a bait that is left in the environment? (Contacts/bait in environment/day) | 0.00002 | 0.000002 | 0.002 |
| What is the probability that a human who makes direct contact with a found bait will contaminate a mucosal membrane? (e.g. an open wound) | 0.002 | 0.001 | 0.003 |
| What is the probability that a human who makes direct contact with a found bait will get the vaccine inside their body (through nose, mouth, etc.)? | 0.00002 | 0.000002 | 0.002 |

| | | | |
|---|---|---|---|
| What percent of the human population will be licked each day by the target animal (Percent licked per year/365)? | 0.002778 | 0.0003 | 0.03 |
| What percent of the human population will be licked each day by the non-target animal (Percent licked per year/365)? | 0.00002 | 0.000002 | 0.002 |
| What is the probability that a human who is licked by an animal will receive the lick on a mucosal membrane (e.g. open wound, mouth)? | 0.002 | 0.0002 | 0.02 |
| What percent of the human population will be bitten each day by the target animal (Percent bitten per year/365)? | 0.003044 | 0.0015 | 0.0045 |
| What percent of the human population will be bitten each day by the non-target animal (Percent bitten per year/365)? | 0.00002 | 0.000002 | 0.002 |
| What proportion of rabid target animals will bite a human before it dies? | 0.4 | 0.2 | 0.6 |
| What proportion of rabid non-target animals will bite a human before it dies? | 0.002 | 0.0002 | 0.02 |
| Among rabid target animals that bite, how many humans, on average, will the rabid target animal bite per day? | 0.266667 | 0.027 | 2.7 |
| Among rabid non-target animals that bite, how many humans, on average, will the rabid non-target animal bite per day? | 0.002 | 0.0002 | 0.02 |
| What is the probability a bite from an animal will penetrate the cranial or peritoneal cavity? | 0.00002 | 0.000002 | 0.002 |
| What proportion of humans who have physically handled the bait in such a manner that the vaccine touched a mucosal membrane or entered the body will seek care? | 0.5 | 0.05 | 1 |
| What proportion of humans that have been licked on a mucosal membrane (e.g. open wound, mouth) by an animal that had recently consumed the bait will seek care? | 0.7 | 0.07 | 1 |
| What proportion of humans that have been bitten by an animal that has recently consumed the bait will seek care? | 0.8 | 0.08 | 1 |
| What proportion of humans that have received a severe bite (one that penetrates the cranial or abdomen) will seek care? | 0.999 | 0.1 | 1 |
